# Urosepsis Causing Gastric Ischemia: A Rare but Deadly Complication

**DOI:** 10.1155/2019/3682049

**Published:** 2019-09-24

**Authors:** Vijay Jarodiya, Chirag Kher, Sangeetha Nanthabalan, Gunjan Shah

**Affiliations:** ^1^St. Mary Mercy Livonia Hospital, Department of Internal Medicine, Livonia, MI 48154, USA; ^2^St. Mary Mercy Livonia Hospital, Department of Gastroenterology, Livonia, MI 48154, USA

## Abstract

A 70-year-old male presented with abdominal pain and altered mental status. He was found to have sepsis secondary to a urinary tract infection with imaging showing hepatic portal venous gas and gastric pneumatosis. Esophagogastroduodenoscopy revealed gastric ischemia extending to the midbody with necrosis and biopsies confirming ischemia. The patient was treated conservatively with intermittent nasogastric tube suctioning, acid suppression therapy and broad-spectrum antibiotics. The patient improved clinically and repeat imaging and EGD showed resolution of the ischemia. The patient's diet was advanced and he was discharged to a long-term acute care facility. Gastric ischemia is a rare condition caused by local or diffuse vascular insufficiency. Management is either surgical or conservative with acid suppression, nasogastric tube suctioning and broad-spectrum antibiotics. Gastric ischemia is often diagnosed late and can have complications such as gastric perforation which carries high morbidity and mortality.

## 1. Introduction

Given the rich vascular supply of the stomach with multiple collaterals, gastric ischemia is a rare phenomenon while indicating a poor prognosis. Gastric ischemia is rarely confirmed histologically and delays in diagnosis or treatment can be deadly. Gastric ischemia can be caused by diffused or localized vascular insufficiency which can be seen in sepsis, thromboembolism or acute thrombosis, local vasculitis, gastric volvulus or gastric dilatation however many cases are idiopathic with no clear etiology. Gastric ischemia is usually managed conservatively with gastric acid suppression, decompression with a nasogastric tube. Antibiotics can also be used to prevent infections for patients at risk for perforation. Surgical options are usually left for patients that have failed conservative management, but patients with perforation, severe necrosis or need for revascularization require immediate surgical intervention. Here we discuss the case of a nursing home patient that presented with nonspecific abdominal pain and altered mental status.

## 2. Case Presentation

**Figure 1:**
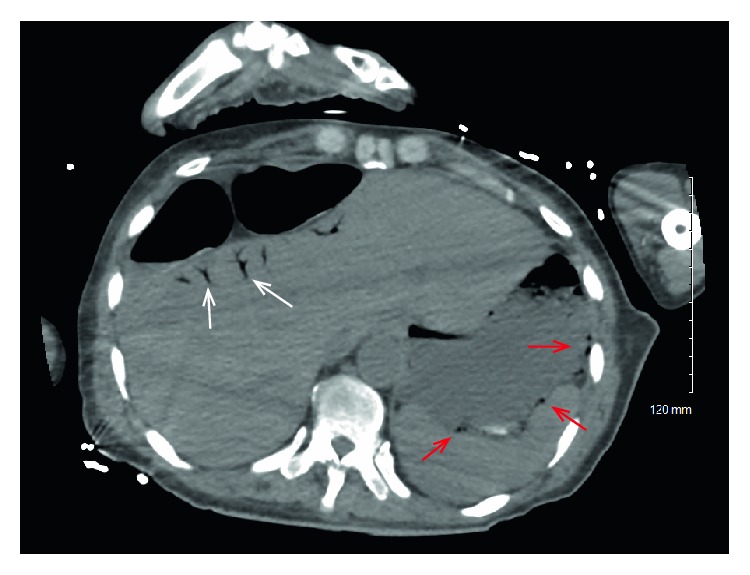
CT findings revealing hepatic portal venous gas (white arrows) and gastric pneumatosis (red arrows).

A 70-year-old male with PMH of CML in remission, peripheral arterial disease on apixaban, hypothyroidism, COPD, HLD, gout, a chronic indwelling catheter who was chronically bedbound presented from a skilled nursing facility with altered mental status and abdominal pain. While in the emergency department, he was hypotensive with an initial blood pressure of 96/53 mmHg and tachycardic. Patient's chronic Foley catheter appeared blocked and upon removal of the catheter purulent drainage was noted. Initial laboratory testing was significant for leukocytosis of 43,400 cells/mL, lactic acidosis with initial lactic acid of 2.3 mMol/L and an acute kidney injury with a creatinine of 2.92 mg/dL and an elevated INR of 2.3, which was secondary to apixaban therapy. The patient's blood pressure did not improve after 30 mL/kg of fluid resuscitation and patient was started on a vasopressor infusion. CT abdomen pelvis without contrast performed in the emergency department revealed gastric pneumatosis with adjacent left upper quadrant portal venous gas along with branching portal venous gas throughout the liver (Figure 1). The patient was started on broad-spectrum antibiotics with cefepime, vancomycin and meropenem and urine cultures, as well as blood cultures, were sent. The patient was transferred to the step-down unit for closer observation.

**Figure 2:**
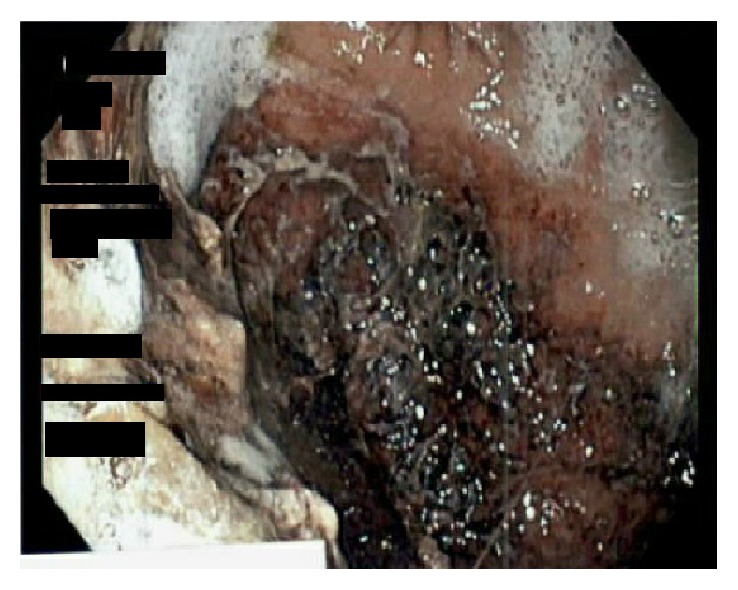
Findings of gastric necrosis and ischemia seen on esophagogastroduodenoscopy.

**Figure 3:**
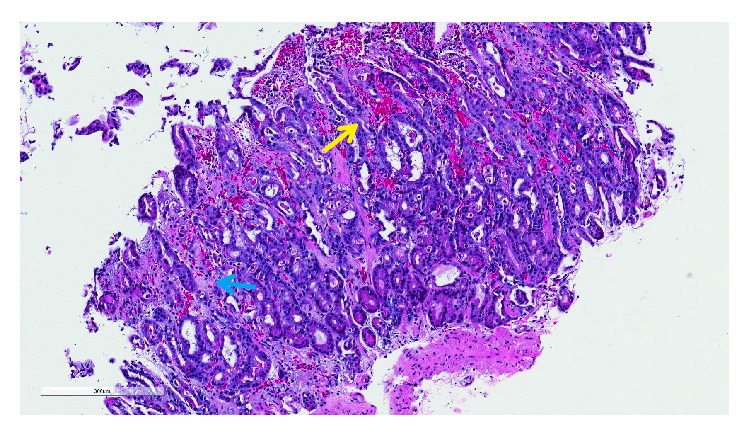
Histologic imaging of gastric biopsy showing areas of hemorrhage (yellow arrow) with mucosal coagulative necrosis (blue arrow), which can be early gastric ischemic changes.

The general surgery and gastroenterology teams were consulted for further evaluation while the patient was in the step-down unit. Given the patient's therapeutic INR and lack of any small bowel findings on the initial CT scan, embolism causing mesenteric ischemia was thought to be unlikely. However given the acute renal failure, CT angiography was not performed. Esophagogastroduodenoscopy was performed revealing proximal stomach ischemia from the midbody to the fundus with some necrosis with normal appearing distal stomach and duodenum (Figure 2). Biopsies revealed acute hemorrhagic gastritis secondary to ischemia with recommendation of surgical resection (Figure 3). The patient was deemed to not be a surgical candidate by the general surgery service given his multiple comorbidities and was managed conservatively with intravenous acid suppression therapy, intermittent nasogastric tube suction, bowel rest with total parenteral nutrition along with broad-spectrum antibiotic therapy for his urosepsis. Patient's blood and urine cultures grew extended-spectrum beta-lactamase Proteus mirablis.

Days later after the patient's acute renal failure resolved, CT angiography was performed which revealed mild narrowing of the celiac artery with patent flow. The superior and inferior mesenteric arteries, as well as the left gastric artery, were all found to be patent and the prior noted gastric pneumatosis and portal venous gas were resolved. The patient began to show clinical improvement and repeat EGD 1 week later revealed gastritis in the proximal stomach with healing pink mucosa and no signs of ischemia or necrosis. The patient was then slowly started on an oral diet and after completion of his antibiotic course was transferred to the medical floor and later discharged to a long-term acute care facility.

## 3. Discussion

Gastric ischemia is a rare clinical entity that is not frequently described in medical literature. Gastric ischemia is a serious condition with estimated mortality rates of an estimated 30% thirty-day mortality and reports of up to a 40% delayed 1-year mortality rate, in part due to delays in diagnosis leading to development of severe complications [1, 2]. It is a rare phenomenon with limited cases reported in the literature, typically caused by either local or diffused vascular insufficiency [3]. Identified causes of gastric ischemia include gastric volvulus [4], gastric dilatation [5], vasculitis [3], disseminated intravascular coagulation and shock [6]. Some cases of gastric ischemia are also iatrogenic, such as after endoscopic submucosal dissection [7], epinephrine injections for nonvariceal upper gastrointestinal bleeding [8, 9], and trans-arterial chemoembolization for hepatocellular cancer [10]. A majority of cases occur at the greater curvature of the gastric body, the posterior wall and the fundus, regions where arterial branches anastomose. The cardia, antrum, lesser curvature, and anterior gastric wall can also become ischemic but are less common sites.

The diagnostic workup for gastric ischemia includes imaging, either noncontrast CT scans or CT angiography. Suggestive findings on imaging include gastric wall pneumatosis and portal venous gas, with estimates of 30–50% of patients having radiologic signs of gastric ischemia [2, 3]. Although these findings are commonly present, they are not specific for gastric ischemia as these signs can be seen with bacterial infections or increased intragastric pressures. CT imaging can also be diagnostic of possible underlying etiologies in patients with ischemia, being able to detect obstructions as well as possible thrombi or emboli on angiography. A negative CT scan also does not rule out gastric ischemia as imaging can be negative, especially in the early phases [2, 3].

Endoscopy is the gold standard for diagnosis and is able to detect early ischemic changes. Mucosal changes that can be seen on endoscopy include mucosal congestion, erythematous or a purple hue of the gastric mucosa, large ulcerations as well as frank necrosis with eschar [2]. Biopsies may reveal capillary dilatation, mucosal edema, vascular congestion or superficial surface erosions as signs of early ischemic changes. Ongoing ischemia is seen histologically with hyalinization and fibrosis of lamina propria, hemorrhagic gastritis and withered atrophic glandular epithelium [3].

Gastric ischemia can be managed conservatively or surgically. Conservative management includes acid suppression and gastric decompression. Gastric ischemia impairs gastric motility causing stomach dilatation which exacerbates ischemia and causes stagnation of gastric acid secretions. These secretions can result in mucosal injury, which can be relieved with nasogastric tube decompression. As a majority of cases have been seen in the dependent parts of the stomach such as the greater curvature and funds, acid suppression helps reduce the chances of acid pooling in the stomach which can cause further ulceration and worsen the ischemia. Patients with ischemia secondary to sepsis or with signs seen on imaging are at increased risk for perforation and should also receive broad-spectrum antibiotics [2, 6]. Surgical intervention is indicated in patients who have a surgically reversible cause of ischemia such as a gastric volvulus, patients who failed conservative therapy, or had complications such as refractory bleeding or full-thickness necrosis and perforation which carry increased morbidity and mortality [2].

Our case highlights a rare cause of gastric ischemia caused by diffuse vascular insufficiency secondary to urosepsis while emphasizing the importance of rapidly identifying and starting treatment for gastric ischemia. Some difficulties in our patient included the vague nature of his presenting complaints with multiple possible causes for his ischemia. Given his history of peripheral arterial disease, arterial emboli could alternatively have caused his gastric ischemia and given the acute renal failure, CT angiography was delayed numerous days until the patient's kidney function improved. Early detection and management of the ischemia led to more rapid improvement of the patient's ischemia: CT angiography 4 days later showed resolved pneumatosis and portal venous gas and repeat endoscopy 7 days later revealed healing mucosa with no signs of ischemia or necrosis.

Gastric ischemia is a rare but potentially fatal condition with an overall poor prognosis. Early diagnosis with imaging or endoscopy and treatment with gastric decompression, acid suppression and broad-spectrum antibiotics or surgical intervention are instrumental in preventing complications such as full thickness gastric necrosis and perforation which carry high morbidity and mortality.

## References

[1] Elwir S., Shaukat A., Mesa H., Colbach C., Dambowy P., Shaw M. (2016). Ischemic gastritis: a multicenter case series of a rare clinical entity and a review of the literature. *Journal of Clinical Gastroenterology*.

[2] Sharma A., Mukewar S., Chari S. T., Wong L. M. (2017). Clinical features and outcomes of gastric ischemia. *Digestive Diseases and Sciences*.

[3] Tang S.-J., Daram S. R., Wu R., Bhaijee F. (2014). Pathogenesis, diagnosis, and management of gastric ischemia. *Clinical Gastroenterology and Hepatology*.

[4] Peterson C. M., Anderson J. S., Hara A. K., Carenza J. W., Menias C. O. (2009). Volvulus of the gastrointestinal tract: appearances at multimodality imaging. *Radiographics*.

[5] Sahoo M. R., Kumar A. T., Jaiswal S., Bhujabal S. N. (2013). Acute dilatation, ischemia, and necrosis of stomach without perforation. *Case Reports in Surgery*.

[6] Patel R. M., DeSoto-LaPaix F., Mallaiah L. R. (1983). Gastric infarction: a complication of endocarditis due to Staphylococcus aureus. *Journal of Clinical Gastroenterology*.

[7] Probst A., Maerkl B., Bittinger M., Messmann H. (2010). Gastric ischemia following endoscopic submucosal dissection of early gastric cancer. *Gastric Cancer*.

[8] Hilzenrat N., Lamoureux E., Alpert L. (2003). Gastric ischemia after epinephrine injection for upper GI bleeding in a patient with unsuspected amyloidosis. *Gastrointestinal Endoscopy*.

[9] Kim S. Y., Han S.-H., Kim K. H. (2013). Gastric ischemia after epinephrine injection in a patient with liver cirrhosis. *World Journal of Gastroenterology*.

[10] Morante A., Romano M., Cuomo A. (2006). Massive gastric ulceration after transarterial chemoembolization for hepatocellular carcinoma. *Gastrointestinal Endoscopy*.

